# Ovarian Cancer Cells in Ascites Form Aggregates That Display a Hybrid Epithelial-Mesenchymal Phenotype and Allows Survival and Proliferation of Metastasizing Cells

**DOI:** 10.3390/ijms23020833

**Published:** 2022-01-13

**Authors:** Sonia Capellero, Jessica Erriquez, Chiara Battistini, Roberta Porporato, Giulia Scotto, Fulvio Borella, Maria F. Di Renzo, Giorgio Valabrega, Martina Olivero

**Affiliations:** 1 Candiolo Cancer Institute, FPO-IRCCS, 10060 Candiolo, Italy; sonia.capellero@ircc.it (S.C.); jessica.erriquez@ircc.it (J.E.); roberta.porporato@ircc.it (R.P.); giulia.scotto@ircc.it (G.S.); mariaflavia.direnzo@unito.it (M.F.D.R.); martina.olivero@ircc.it (M.O.); 2 Department of Oncology, University of Torino, 10129 Torino, Italy; 3 Unit of Gynaecological Oncology Research, European Institute of Oncology, IRCCS, 20100 Milan, Italy; chiara.battistini@ieo.it; 4 Gynecology and Obstetrics 1, Department of Surgical Sciences, City of Health and Science, University of Turin, 10100 Turin, Italy; fulvio.borella@unito.it

**Keywords:** ovarian cancer, ascites, PAX8, αSMA, fibronectin, epithelial-mesenchymal phenotype

## Abstract

Peritoneal metastases are the leading cause of morbidity and mortality in ovarian cancer. Cancer cells float in peritoneal fluid, named ascites, together with a definitely higher number of non neo-neoplastic cells, as single cells or multicellular aggregates. The aim of this work is to uncover the features that make these aggregates the metastasizing units. Immunofluorescence revealed that aggregates are made almost exclusively of ovarian cancer cells expressing the specific nuclear PAX8 protein. The same cells expressed epithelial and mesenchymal markers, such as EPCAM and αSMA, respectively. Expression of fibronectin further supported a hybrid epithelia-mesenchymal phenotype, that is maintained when aggregates are cultivated and proliferate. Hematopoietic cells as well as macrophages are negligible in the aggregates, while abundant in the ascitic fluid confirming their prominent role in establishing an eco-system necessary for the survival of ovarian cancer cells. Using ovarian cancer cell lines, we show that cells forming 3D structures neo-expressed thoroughly fibronectin and αSMA. Functional assays showed that αSMA and fibronectin are necessary for the compaction and survival of 3D structures. Altogether these data show that metastasizing units display a hybrid phenotype that allows maintenance of the 3D structures and the plasticity necessary for implant and seeding into peritoneal lining.

## 1. Introduction

Epithelial ovarian cancer (EOC) is the deadliest gynecological malignancy in women, being diagnosed at advanced stages in most instances [1]. Unlike most carcinomas, EOC very rarely spreads through the bloodstream and preferably disseminates into the peritoneum [2], giving rise to ascites, i.e., accumulation of abdominal fluid with floating cancer cells, and peritoneal nodules. Actually, ascites is present in more than one-third of ovarian cancer patients at diagnosis and in almost all cases of relapse [3,4]. Dissemination into the peritoneum is mediated by shedding of tumor cells from the primary site [2,5] and/or by reseeding of peritoneal metastases [6]. Ovarian cancer cells break away from the primary or secondary tumor masses individually or as cell-cell aggregates [7].

Multicellular aggregates in ascites, also called spheroids, are crucial for metastatic dissemination of ovarian cancer. First, spheroids are abundant in patients with advanced disease and correlation has been reported between resistance to platinum-based chemotherapy and percentage of spheroids in patients [8]. Moreover, several model systems, based on experimental aggregates of established cell lines or purified cell populations have allowed showing that ovarian cancer cells forming 3D structures are protected from anoikis and chemotherapeutics, preserve cancer cell stemness, attach to and clear the mesothelial lining of the peritoneum, adhere to the underlining extra cellular matrix (ECM) and eventually form metastatic noduli (for reviews see [3,9]). Again using 3D models, these abilities have been linked to the expression of a variety of proteins, such as N-cadherin [10], autocrine-secreted fibronectin [11,12], integrins [13] and an array of mesenchymal markers [14,15].

The cellular composition of whole ascites varies across patients, in some instances being tumor cells up to less than 1% of the whole ascites volume [4]. Izar et al. [16] carried out single cell RNA sequencing of cells of ascites from EOC patients and showed that only approximately 7.9% expressed bona fide ovarian cancer-associated markers, such as EPCAM and CD24. Indeed, EOC ascites has been described as composed by malignant and non-malignant cells, the latter in turn identified as cancer-associated fibroblasts [13,17], mesothelial cells [18,19], macrophages [20] and leukocytes [13].

Given the relatively variable frequency of spheroids in diverse ascites, it was likely that cells composing spheroids were not picked up thoroughly by RNAseq protocols. Thus, we hypothesized that spheroids show a more specific phenotypic pattern possibly associated to their ability to form metastases. Therefore, we studied spheroids from EOC patients’ ascites using a minimal manipulation and surprisingly show here that they are all composed by almost only ovarian cancer cells; these cells display a full epithelial phenotype but also a partial mesenchymal transition consisting of the expression of alpha smooth muscle actin (αSMA) and fibronectin (FN1) secretion, critical for the formation and survival of 3D structures formed by ovarian cancer cells.

## 2. Materials and Methods

### 2.1. Collection of Samples

Between 2019 and 2020, we collected 23 ascites samples of epithelial ovarian carcinoma EOC from 15 patients at paracentesis carried out as palliative care management. Samples were collected according to the “Profiling” Protocol approved by the local Ethical Committee (initially approved on 2 March 2011 n° 4/2011 by Comitato Etico Interaziendale AOU San Luigi and then on 14 October 2020 n° 296/2020, v.8.0, approved by the competent Ethical Committee of Istituto di Candiolo). Informed consent subscribed by enrolled patients is therefore compliant to the standards set by the Declaration of Helsinki. Only ten out of 23 samples, collected from six patients, were suitable for phenotyping as the others were highly hemorrhagic.

Fresh abdominal effusion fluids were centrifugated at 1000 RPM; cell pellet was washed in PBS and immediately red blood cells were lysed with BD Pharm Lyse™ (BD Biosciences); then cells were washed once in basal culture media (RPMI 1640, Gibco™). Part of bulk population was immediately fixed in buffered formalin 10% (Sigma) embedded in Histogel™ (Thermo Scientific^®^), while the rest of the sample was allocated for in vitro experiments.

### 2.2. Cell Lines

Commercially available ovarian cancer cell lines: (OVCAR-4, A2780, OVCAR-8, IGROV-1, COV 362, OV-90) and human fibroblast (BJ-5ta CRL-4001™) were obtained from American Type Culture Collection (ATCC). All cell lines have been characterized and maintained as suggested by the provider.

### 2.3. Immunofluorescence

Ovarian cancer spheroids were characterized with immunofluorescence using 5 μm thick sections cut from formalin-fixed paraffin-embedded samples, mounted on slides and treated following standard procedures. Tissue sections were deparaffinized with absolute xylene and rehydrated with decreasing concentrations (100%, 70%, 50%) of ethyl alcohol. Antigen retrieval was performed by boiling the sections in citric acid, at pH 6, in microwave (750 W, twice for 5 min) and, after room temperature cooling, they were treated with Dako Protein Block/PBS 0.1% Tween-0.3% Triton-1% BSA for 30 min in a moisture chamber at room temperature. Direct antibodies were mixed in Dako Antibody Diluent with Background Reducing Components according to the manufacturer’s protocols and slides were incubated in a closed humid chamber at 4 °C overnight, with different conjugated primary antibodies. Subsequently, nuclei were stained with DAPI and slides were mounted with SI PREP, AQUA-MOUNT (Thermo Fisher Scientific, Waltham, MA, USA, cod.APTA-125-AM).

To perform IF on adherent cells, cell lines were plated in a 6-well plate on glass coverslips coated with 0.1% gelatin from porcine skin (Sigma-Aldrich, Saint Louis, MO, USA, #G9136) and allowed to adhere overnight. Cells were washed in PBS, fixed in 4% paraformaldehyde (PFA), permeabilized with absolute methanol for 15 min at 4 °C and saturated with Dako Protein Block/PBS 0.1% Tween-0.3% Triton-1% BSA for 30 min, then incubated overnight with different conjugated primary antibodies as previously described. Nuclei were stained with DAPI and glass coverslips were mounted with SI PREP, AQUA-MOUNT (Thermo Fisher Scientific, cod.APTA-125-AM).

When necessary, commercial ovarian cancer cell lines and ascites pellets were embedded in growth factor–reduced Matrigel (Tissue culture supplement, Corning^®^ Matrigel^®^, 734-0272) and plated in μ-Slides 8 well imaging chambers (80826, Ibidi GmbH). A total of 5000–8000 cells were approximately dispersed in 15 μL droplet of Matrigel (final concentration of Matrigel 75%). Once the Matrigel was solidified, 200 μL of appropriate culture medium was added to each well. After 5 days cell culture medium was removed and Matrigel drops were washed in PBS, fixed in 4% PFA, permeabilized with absolute methanol, saturated and incubated with different conjugated primary antibodies as previously described. Nuclei were stained with DAPI and 200 μL of PBS was added to each well. When necessary, 3D spheroids were cultivated in presence of PND-1186 (VS-4718) for 5 days.

Immunofluorescence was carried using the following conjugated primary antibodies selected from the CyCIF list (website https://www.cycif.org (accessed on 29 November 2021)): EpCAM (VU1D9) Mouse MAb (AlexaFluor^®^ 488 Conjugate, Cell Signalling Technology#5198); βactin (13E5) Rabbit MAb (AlexaFluor^®^ 555 Conjugate, Cell Signalling Technology, #8046); Ki-67 (D3B5) Rabbit MAb (Alexa Fluor 488 Conjugate, Cell Signalling Technology, #11882); anti-CD11b rat MAb [M1/70] (Alexa Fluor^®^ 488, abcam, ab197701); anti-CD163 rabbit MAb [EPR14643-36]-C-terminal (Alexa Fluor^®^ 647, abcam ab218294); anti-Fibronectin rabbit MAb [F1] (Alexa Fluor^®^ 488, abcam, ab198933); anti-PAX8 rabbit antibody [EPR18715] (Alexa Fluor^®^ 647, abcam, ab215953); anti-alpha smooth muscle actin rabbit MAb [EPR5368] (Alexa Fluor^®^ 555, abcam ab202509); CD45RB rabbit MAb Monoclonal Antibody (PD7/26), Alexa Fluor 488 (eBioscience, #53-9458-82); DAPI (Thermo Fisher Scientific, 62248). Unconjugated primary αSMA antibody was Clone 1A4 (Dako, #M0851).

### 2.4. RNA In Situ Hybridization and Protein Immunohistochemistry

To evaluate Human ACTA2 expression in ovarian cancer cells, a co-detection assay was used on formalin-fixed paraffin-embedded (FFPE) samples, combined RNAScope 2.5 RED technology (Advanced Cell Diagnostics-a Bio-Techne brand^®^) on ACTA2 mRNA and immunohistochemistry of PAX8 protein. Five μm thick sections of formalin-fixed paraffin-embedded tissue samples, deparaffinized and dehydrated following standard procedures, were pretreated with the following consecutive incubations: (i) 10 min at room temperature with hydrogen peroxide; (ii) about 10 min at boiling temperature with target retrieval maintained in a steamer (in particular: 10′ for #5565 sample and 13′ for #5326 and #5819 samples); (iii) 10 min at 40 °C with protease in ACD HybEZ hybridization oven. To set the optimal condition, a wide range of temperature and time conditions in pretreatment steps with positive and negative probes control were explored. To ensure interpretable results, the assay was performed using in parallel positive and negative controls: the endogenous housekeeping gene was Hs-PPIB (ACD-Biotechne ref. 313901) used as positive control to assess both tissue RNA integrity and assay procedure. The bacterial gene dapB (ACD-Biotechne ref. 310043) was used as negative control to assess background signals. Pretreated samples were incubated for 2 h at 40 °C with appropriate probes (ACTA2 probe ref. 444771 was used concentrated 5×) and then the signal was amplified (1 h AMP5) and twice detected with fast red substrate. The samples were saturated with TBS-T-T+ 5% Normal Goat serum (Vector-D.B.A S-1000) for 1h at room temperature, and primary antibody against PAX8 (PAX-8 (MRQ-50), 760-4618 Roche ref. 760-4618) was incubated in a humid chamber overnight at +4 °C. Secondary antibody anti-mouse-HRP (Dako K-4001) was stained 1 h at room temperature, and the signal has been developed with DAB substrate (Dako K-3468) and counterstained with hematoxylin 50% in water (Sigma GHS1128).

### 2.5. Viability Assay

CellTiter-Glo^®^ assay was used to evaluate the effect of PND-1186 (VS-4718, Selleckchem, #S7653) on ovarian cancer cells proliferation, according to the manufacturer’s protocol (Promega, Madison, WI, USA). Fold increase has been calculated for 0.5 μM and 1 μM of inhibitor concentration and plotted using a GraphPad Prism version 7.02 (San Diego, CA, USA).

### 2.6. Image Acquisition and Quantification

Images of fixed cells were acquired with Leica TCS SPE II confocal microscope, 40× of magnification and were analyzed with NIH ImageJ (W. Rasband, NIH) software. To obtain the percentage of tumor cells per field, we divided the PAX8 positive area by the DAPI positive area and we statistically estimated the area occupied by a single nucleus. Since spheroids are very heterogeneous in shape and size, sometimes even within the same sample we decided to score αSMA giving 0 to 3 values, that indicate the amount of antibody presence into cells. If the score is 0 to 1+, the sample was slightly positive; if the score was 2+, the sample was considered positive; if the score was 3+ the cells were considered highly positives. Epifluorescent 4× and 40× images of 3D spheroids were collected using the inverted Ti2 Eclipse microscope of LIPSI (Nikon) and analyzed by NIS-Elements (Nikon) software. To estimate dimension distributions of aggregates formed by ovarian cancer cells and of the spheroid of representative ascite in presence of the PDN-1186 FAK inhibitor we used NIS-Elements software to calculate Feret diameter in 4× images of 2 different experiments.

## 3. Results

### 3.1. Spontaneous Spheroids of EOC Patients’ Ascites Show Both Epithelial and Partial Mesenchymal Phenotype

We studied in depth ten samples of EOC ascites from six patients. Indeed, longitudinal samples of ascites were obtained from successive paracenteses of patients at relapse in palliative care setting. Three out of these six patients were not previously treated with chemotherapy. Patients’ characteristics are reported in Supplementary Table S1.

Manipulation of the samples was minimal to avoid selection of cell subpopulations. After collecting cells from whole ascites and brief washing with gentle centrifugations, the bulk cell population was immediately fixed in buffered formalin, embedded in Histogel™ and then in paraffin.

**Figure 1:**
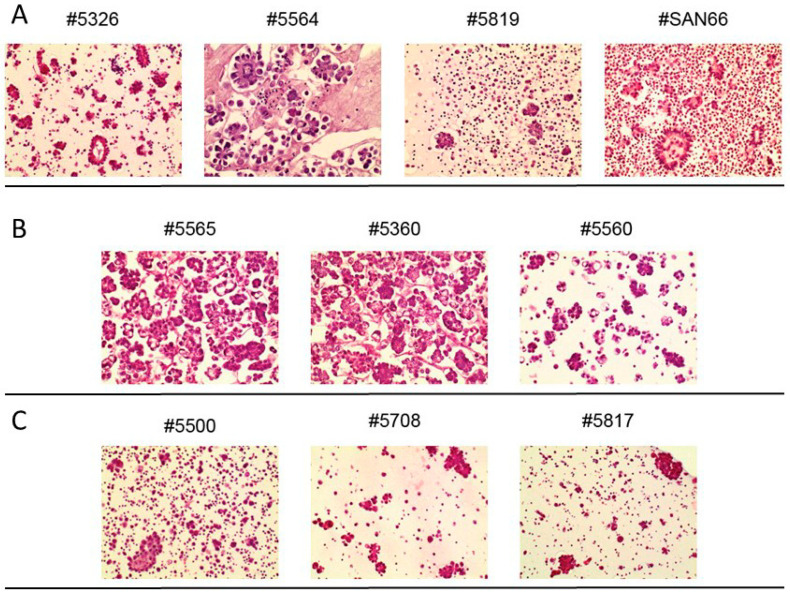
H&E staining of spheroids from different patients’ paracenteses (**A**) and three longitudinal samples of ascites collected from one relapsed patient (**B**) and from another relapsed patient (**C**). Numbers on top of each panel correspond to the Profiling protocol numbers of the collected ascites. Details of each #case are in Supplementary Table S1. 20× magnification.

Figure 1 shows representative H&E-stained sections of ascites samples. As already known [3,17], spheroids in ascites vary in frequency, size and structure.

**Figure 2:**
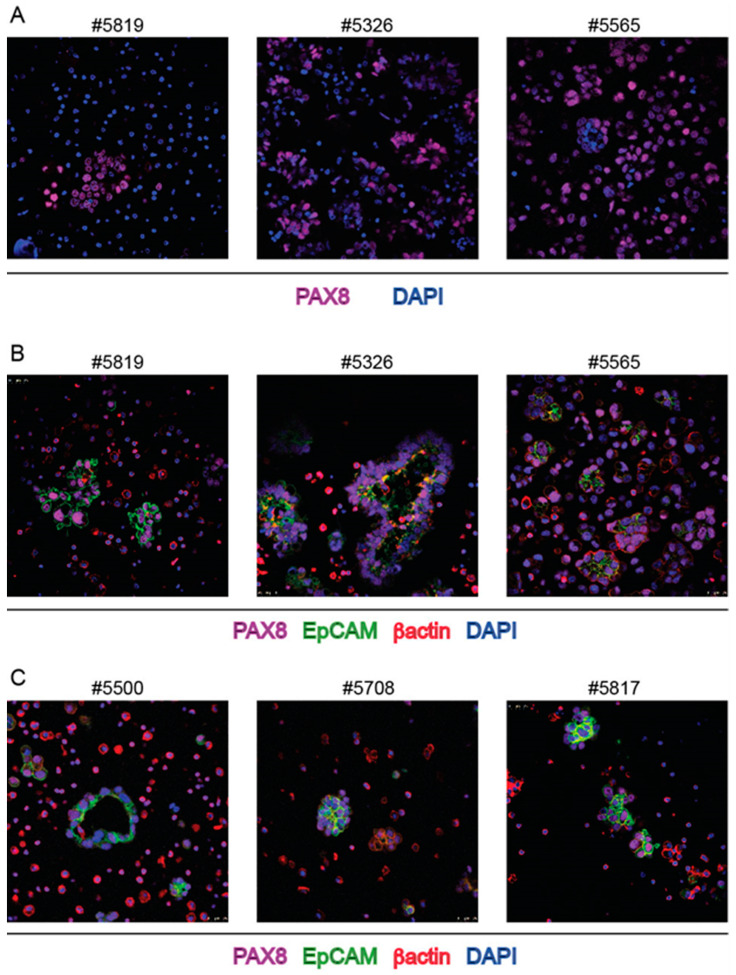
IF staining of spheroids from bulk ascites samples with PAX8, EpCAM and βactin MAbs. (**A**,**B**): three independent cases stained with the indicated MAbs; (**C**): ascites samples from three successive withdrawals of ascites of the same patient stained with the indicated MAbs. Numbers on top of each panel correspond to the Profiling protocol numbers of the collected ascites. Details of each #case are in Supplementary Table S1.

Immunofluorescence (IF) was used to characterize cell populations composing ascitic spheroids. Regardless of the shape and size, spheroids came out to be totally constituted by PAX8 positive cells, i.e., cells with nuclear localization of the marker of HGS-EOC. Representative images are shown in Figure 2.

PAX8 is a paired-box gene important in embryogenesis of the thyroid, Müllerian, and renal/upper urinary tracts, found increased in a number of carcinomas derived from these tissues and namely in approx. 100% of HGS-EOCs (see e.g., [21]). Conversely, both mesothelial cells and mesothelioma show PAX8 negative expression [22], so that PAX8 staining reliably distinguishes ovarian serous tumors from malignant mesotheliomas [21,23]. More importantly, PAX8 is a key gene in HGS-EOC, as in experimental settings it drives the transformation of the fallopian tube epithelium, from which HGS-EOCs derive [24]. We did not extensively use in this study WT1 previously described as HGS-EOC as marker, as it is also expressed by mesothelial cells [25]. Quantification of PAX8 positive cells compared to all cells stained with DAPI showed that in the whole ascites the percentage of HGS-EOC cells varied from less than 2% to 70% in the sample with the highest cellularity (Supplementary Figure S1), in line with data reported by other Authors (for a review see [4]). Lack of intra patient variability in our series was shown not only by the analyses of several spheroids from a single ascites sample (see e.g., Figure 2), but also by the analyses of multiple longitudinal and independently obtained samples derived from the palliative care of two patients (Supplementary Figure S1).

To further confirm that ovarian cancer cells are the main component of spheroids, EpCAM (Epithelial Cell Adhesion Molecule) was utilized as an additional marker. Although EPCAM is reported as a membrane protein expressed by any epithelial cell type, its overexpression in carcinomas and in particular by ovarian carcinoma cells is used to distinguish the latter cells from mesothelial and non-epithelial cells in human ascites [26]. As expected, EpCAM antibodies labelled the cell surface of PAX8+ cells in spheroids (Figure 2B,C) with a polarized distribution.

**Figure 3:**
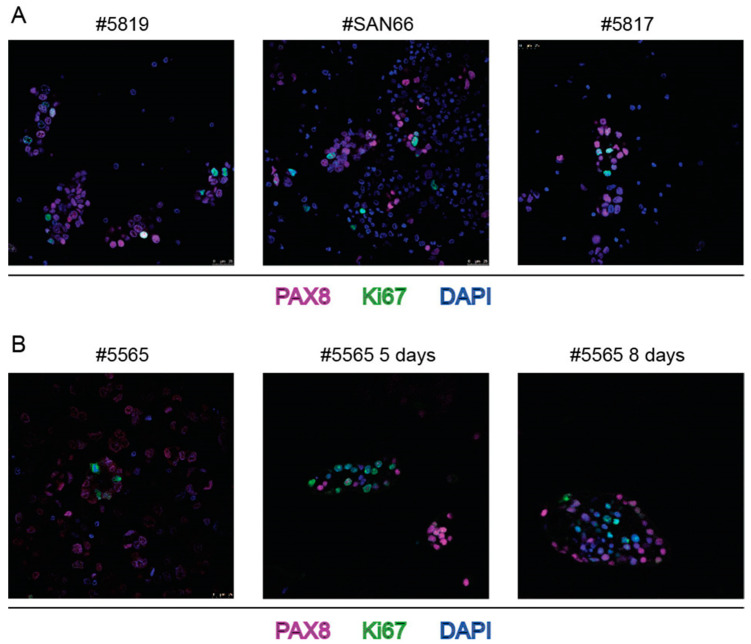
Ki67 staining of spheroids from bulk ascites samples combined with staining with PAX8 MAb and DAPI. (**A**) three independent cases; (**B**): ascites sample #5565 cultivated in Matrigel for 5 and 8 days. Numbers on top of each panel correspond to the Profiling protocol numbers of the collected ascites. Details of each #case are in Supplementary Table S1.

Then, Ki67 antibody was used to assess whether spheroid cells were alive and EOC PAX+ cells were proliferating. As shown in Figure 3A a number of PAX8+ cells in spheroids were stained with Ki67 antibody. Moreover, spheroids could be cultivated up to 12 days. Figure 3B shows the increased number of Ki67 positive cells after 5 and 8 days of cultivation.

**Figure 4:**
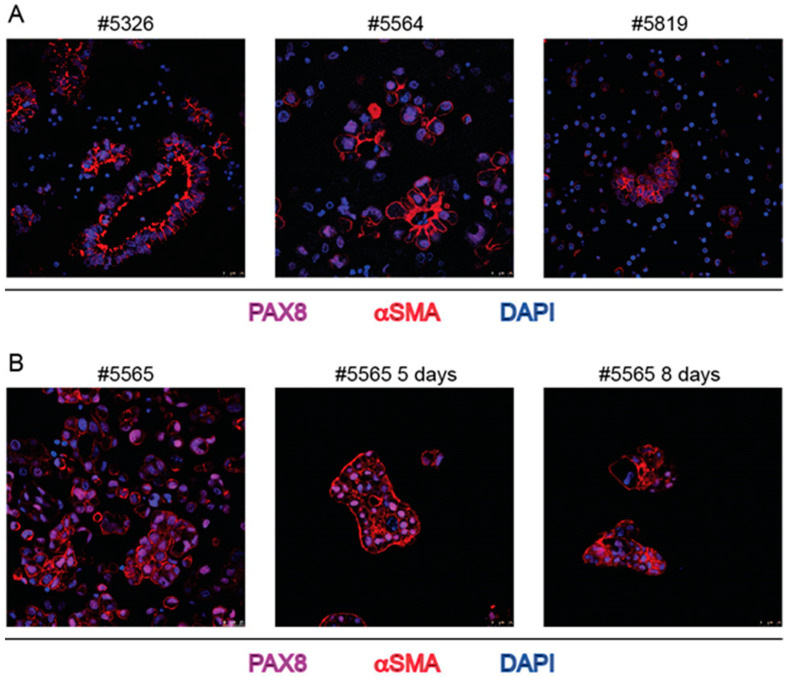
IF staining of spheroids from bulk ascites samples with PAX8 and αSMA MAbs and DAPI. (**A**): three independent cases; (**B**): ascites sample #5565 cultivated in Matrigel for 5 and 8 days. Numbers on top of each panel correspond to the Profiling protocol numbers of the collected ascites. Details of each #case are in Supplementary Table S1.

**Figure 5:**
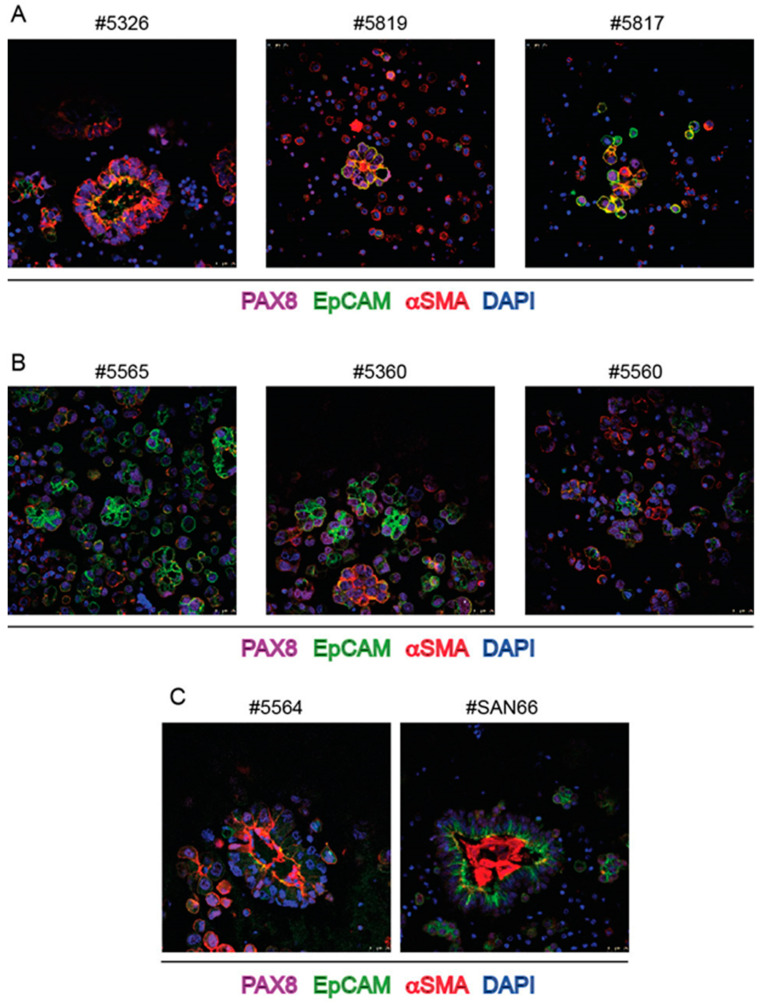
IF staining of spheroids from bulk ascites samples with PAX8, EpCAM and αSMA MAbs and DAPI (**A**): three independent cases; (**B**) three successive withdrawals of ascites of the same patient; (**C**): two independent cases stained with PAX8, EpCAM and αSMA MAbs to show staining of a few αSMA positive cells with PAX8 negative nuclei. Numbers on top of each panel correspond to the Profiling protocol numbers of the collected ascites. Details of each #case are in Supplementary Table S1.

Surprisingly, section staining with αSMA antibody that is commonly used to identify fibroblasts (see control staining in Supplementary Figure S2A) marked a sub-membrane moiety of ovarian cancer cells, mainly at the inner surface of spheroids (Figure 4), in most instances evidently overlaying the EpCAM staining (Figure 5A,B).

**Figure 6:**
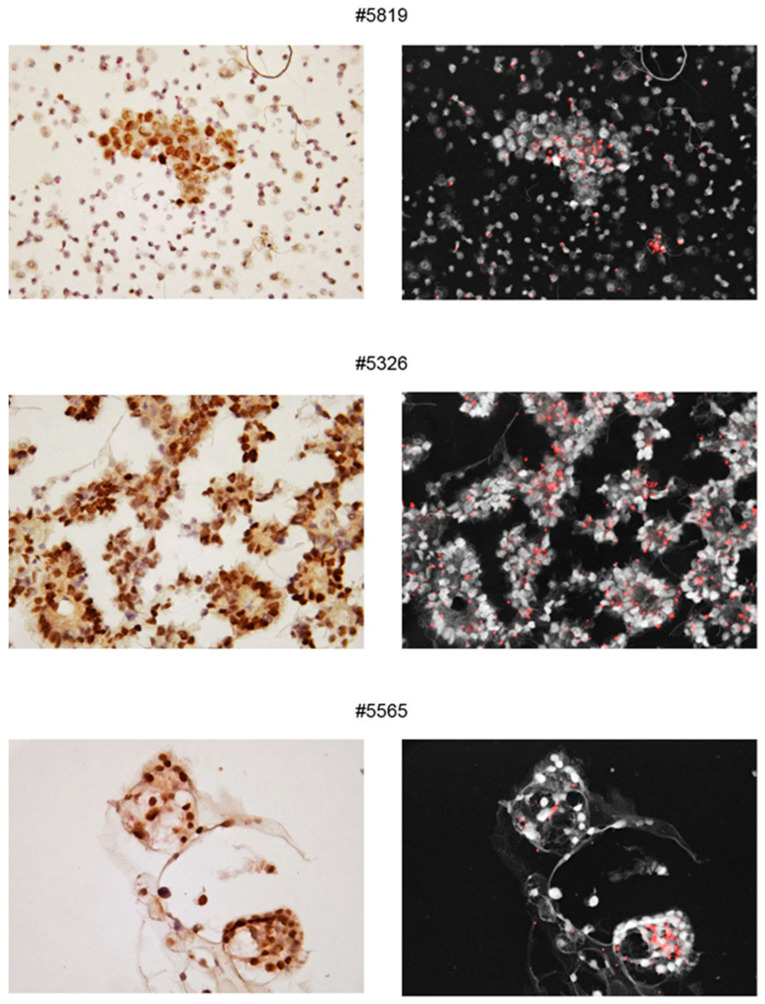
RNAscope showing ACTA2 transcripts (red dots in panels on the left, better visible as fluorescent dots in panels on the right) encoding αSMA in the epithelial cells of bulk ascites samples. Cells are stained with primary PAX8 MAb followed by HRP and DAB substrate and nuclei were counterstained with hematoxylin. Numbers on top of panels correspond to the Profiling protocol numbers of the collected ascites. Details of each #case are in Supplementary Table S1.

The expression of αSMA was maintained not only for spheroids of the same patient derived from successive withdrawals (Figure 5B) but also after spheroid cultivation (Figure 4B). To rule out possible unspecific cross reactions with βactin, another αSMA-specific antibody, similarly generated against the NH2-terminal sequence of the protein was used and found to stain the same moieties in the cells (Supplementary Figure S2B). Again, to rule out possible unspecific cross-reaction, βactin antibody was used and found to stain different cells and different subcellular structures in spheroids (see Figure 2). More importantly, RNAscope with a sequence specific probe confirmed that the bona fide epithelial ovarian cancer cells, stained with PAX8 MAb, expressed αSMA RNA encoded by the ACTA2 gene (Figure 6). Only in ascites of two patients and in these two cases only in a few large spheroids bona-fide fibroblasts were likely detectable, i.e., spindle-shaped cells stained with αSMA antibody and with a central PAX8 negative nucleus (Figure 5C).

**Figure 7:**
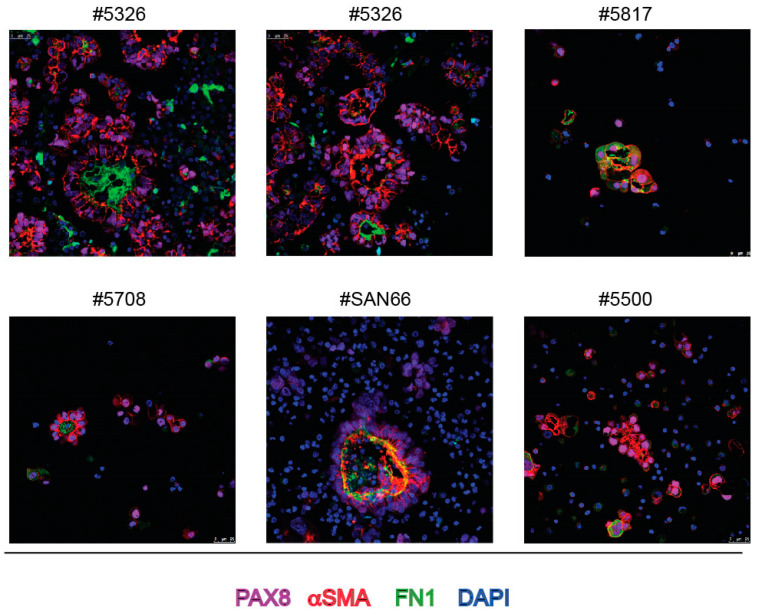
IF staining of spheroids from bulk ascites samples with FN1, PAX8 and αSMA MAbs and DAPI. Intracellular FN1 was detected in samples #5817 and #5500. Z-stack of images of samples #SAN66 is shown. Numbers on top of each panel correspond to the Profiling protocol numbers of the collected ascites. Details of each #case are in Supplementary Table S1.

Given the widespread staining with αSMA MAb but also with EpCAM MAb and their polarized localization in spheroids, we hypothesize that ovarian cancer cells in spheroids undergo the so-called “partial” epithelial-to-mesenchymal plasticity (for a review see [27]). This was confirmed by the detection of fibronectin (FN1) in the core of a number of spheroids (Figure 7). It is known that soluble FN1 is detectable in ascitic fluid [28] and also that endogenous FN1 is secreted by ovarian cancer cells [29]. Thus, it was not surprising to detect FN1 staining outside and inside cells (Figure 7).

**Figure 8:**
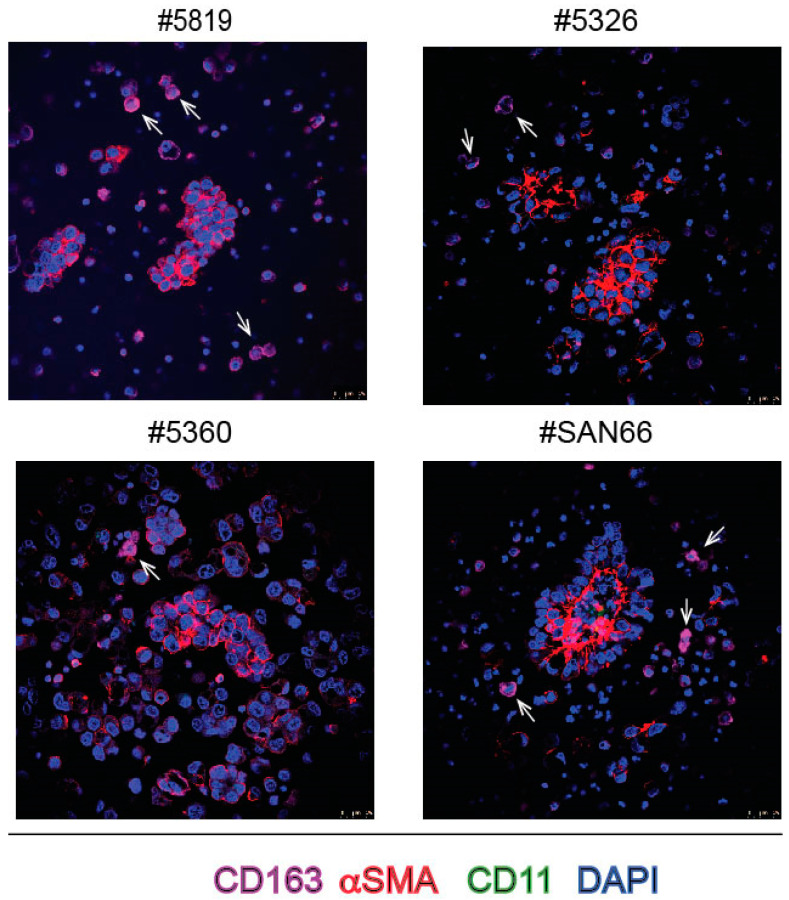
IF staining of spheroids from bulk ascites samples with αSMA MAb and DAPI together with CD11 and CD163 MAbs used as to visualize macrophages. The rare CD163 positive cells are indicated by arrows.

In a thorough single-cell analysis of ascites it had been shown that macrophages are a fundamental component of the ascites ecosystem [16]. We used anti CD11b and anti CD163 MAbs to evaluate the presence of tumor-associated macrophages in ascites spheroids. As shown by Figure 8, in some sections, rare CD163 positive cells were visible, located outside the spheroids, while we detect very rarely CD11 positive cells and only in a few sections, again outside the spheroids (not shown), suggesting that in the ascites M2 state macrophages were more numerous than those in the M1 state and mainly localized outside the spheroids.

CD45 positive cells, i.e., cells of hematopoietic lineages, also were very rarely found in spheroids, but abundantly detectable in the bulk single cell population of ascites (Supplementary Figure S3). This was not surprising as CD45 positive cells are reported to constitute more than 50% of cells in most ascites samples [4].

### 3.2. SMA and Endogenous FN1 Are Necessary for the Formation of 3D Structures by Ovarian Cancer Cells

**Figure 9:**
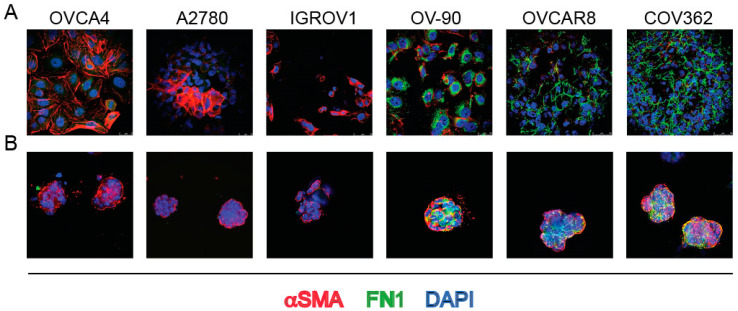
IF staining of the indicated ovarian cancer cell lines with DAPI and αSMA and FN1 MAbs (merged) either grown as monolayer cultures (**A**) or forming 3D aggregates in Matrigel (**B**).

To assess whether the mesenchymal-like phenotype is critical for the formation of 3D structures by ovarian cancer cells, we selected ovarian cancer cell lines with different expression levels of αSMA and FN1, based on the expression of the two specific mRNAs, as reported in the database of Cancer Cell Line Encyclopedia [30]. Immunofluorescence showed that the proteins were differently expressed in agreement with the distribution of mRNAs. Among the six ovarian cancer lines shown in Figure 9A, the OVCAR4 and A2780 cells displayed the highest-level expression of αSMA and the lowest of FN1, while the COV362, OVCAR8 and OV90 cells showed the opposite pattern of protein expression, being αSMA barely detectable in the latter cell lines. When the above-listed cell lines were allowed to form organoid-like 3D structures in Matrigel, submembrane expression of αSMA was found in all cell lines (Figure 9B).

**Figure 10:**
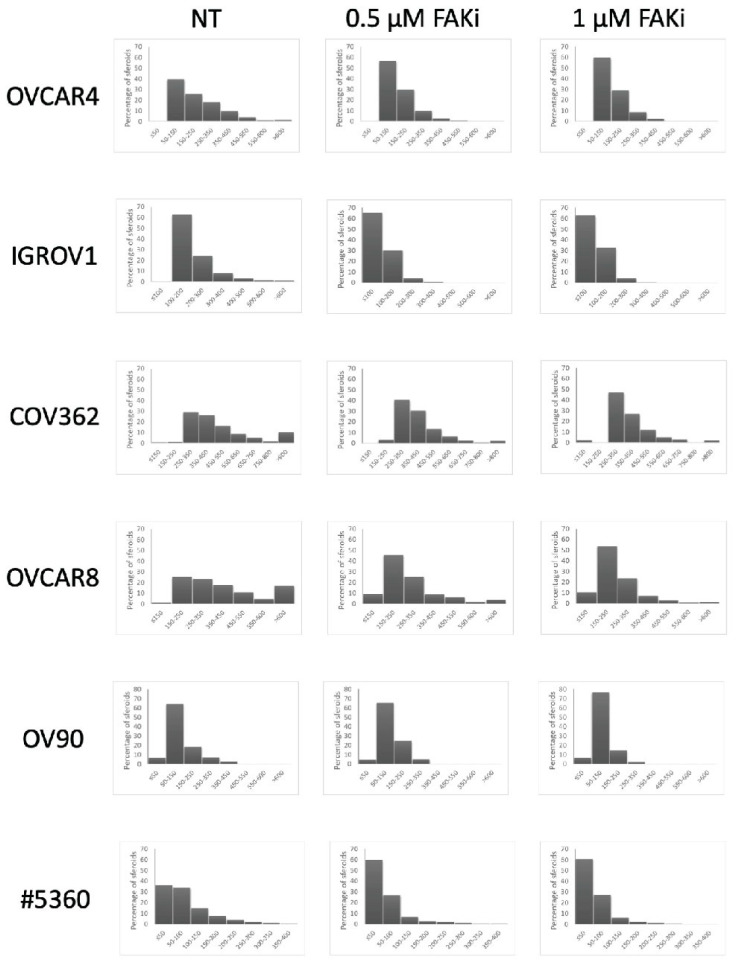
Feret diameter of the 3D aggregates formed by the listed ovarian cancer cell lines and of the spheroid of the bulk ascites sample #5360 grown for 5 days in the presence of the PDN-1186 FAK inhibitor (FAKi) at the indicated concentrations.

Using Ingenuity Pathway Analysis [31] we ascertained that the proline-rich tyrosine kinase 2 (PTK2B/PYK2), which belongs to the family of FAK kinase, regulates the expression of both αSMA and FN1. This is in accordance with previous reports showing the up-regulation of αSMA by this kinase (see e.g., [32]). Less known is the role of FAK upstream fibronectin expression, its role downstream being well known. We cultivated for 5 days the organoid-like structures formed by cell lines in the presence of the highly specific PDN1186 (VS4718) FAK inhibitor (FAKi, ref. [33]). The inhibitor strongly affected the ability of the treated cells to aggregate in organoid-like structures as in the presence of FAKi only small 3D structures were visible (Figure 10), without impairment of cell proliferation (Supplementary Figure S4). Interestingly, the Feret diameter of patient-derived ascites spheroids cultivated for 5 days in the presence of FAKi was also reduced (Figure 10).

## 4. Discussion

Advanced-stage EOC is characterized by early and massive metastatic dissemination in the peritoneum. Ascites, made of fluid and diverse cell types, is the vehicle of dissemination. Different experimental approaches have been used to identify the metastasis-prone cancer cells and assess the contribution of non-malignant cells, as both replenish ascites as single cells and aggregates. Malignant cells of aggregates, here named spheroids, in particular are considered the main source of EOC metastases, as experimental 3D structures have been shown to limit differentiation of EOC cells, i.e., the maintenance of the stem cell phenotype, and to protect cells from death due to loss of anchorage (anoikis) and from attack by chemotherapeutics (for review see [2,9]).

However, the contribution of cancer cells to spheroids was poorly defined. Indeed, spheroids of patients’ ascites are usually described as heterogeneous structures, made of a small number of malignant cells and diverse non-malignant cell types (see e.g., [8,13,34,35]). In addition, the fraction of cancer cells of the whole ascites varies across patients and is reported to contribute from less than 1% [4] to approximately 8% [16] of the total cell population. Finally, the single-cell analyses of ascites showed the abundant presence in ascites of cancer-associated fibroblasts (CAFs) and tumor-associated macrophages (TAMs) [16].

We show here that, minimizing manipulation, we have been able to determine that spheroids from EOC are all almost exclusively made of PAX8+, i.e., epithelial ovarian cancer cells. Rarely we found in spheroids spindle-shaped cells stained with αSMA antibodies, thus identifiable as bona fide CAFs, as reported by other Authors (see e.g., [13]). Similarly, we did not detect either CD45+ in spheroids, while we found several PAX8-/CD45+ cells outside spheroids in the bulk ascites cell preparations. We found a few TAMs and mainly with the M2 phenotype, i.e., cells stained with CD163 MAb, again outside the spheroids. TAMs have been shown to favor peritoneal metastases of ovarian cancer in mouse models [36]. CAF combined with EOC cells in artificial heterotypic aggregation form pro-metastatic units [13]. Altogether, data suggest that CAFs and TAMs rather form the ecosystem of ascites and might contribute to progression and metastases mainly via the secretion of cytokines, such as EGF, IL6 and TGFbeta (see e.g., [15,37]) and thus provide an environment supporting progression, chemoresistance and immune evasion [4].

Additionally, we show here that almost all PAX8+ EOC cells in spheroids express αSMA and most spheroids contain FN1, which are markers usually associated to epithelial-mesenchymal transition (EMT). However, the same spheroid cells maintain the expression of epithelial markers, such as EpCAM, and cell polarization. Thus, all spheroid cells display a hybrid phenotype, also referred to as “partial” EMT or better as epithelial-mesenchymal plasticity (EMP) (for a review see [27]). Interestingly, the thorough scRNAseq analysis reported by Izar et al. [16] showed that EPCAM+/CD24+ cells of the ascites of one out of seven patients analyzed express mesenchymal genes such as the ACTA2 gene encoding for αSMA. The EMP might be necessary for these spheroids to achieve their metastatic potential, as ovarian cancer cells implant into the peritoneum by breaching the mesothelial barrier and contacting the underlining ECM [38]. In line, spheroids formed in vitro by colorectal cancer cells up-regulated expression of αSMA [39]. Moreover, high expression of αSMA marks more malignant breast carcinoma [40].

The hybrid partial mesenchymal phenotype described here in EOC spheroids is also in line with the notion that, in metastasizing carcinoma cells, a complete epithelial to mesenchymal transition is never observed, not only because the reversion of the EMT phenotype (mesenchymal to epithelial transition, MET) would be mandatory but never shown in metastatic site, but also because carcinoma cells always retain the epithelial phenotype (for a perspectives review see [27]). More importantly, while carcinoma cells in ascites, including those forming spheroids, do not show important genetic variation (see e.g., [5,34]), EMT itself is the best example of phenotypic plasticity that cancer cells acquire in several experimental conditions. Therefore, we believe that the morphological description of nonmanipulated spheroids shown here better reflect the actual phenotype of ovarian cancer cells in ascites. EMT has also been frequently associated to metastasis in ovarian cancer (for a review see [10]), but in this cancer, too, the clinical significance of EMT remains controversial, although even in ovarian cancer the association between the cancer stem cell phenotype and EMT has been shown [41]. Data shown here, however, confirm the hypothesis that ovarian carcinoma cells are prone to plasticity. The striking expression of αSMA was unexpected, as only anecdotal reports have described before this expression in HGS-EOC [42,43]. While we show here that α-SMA is necessary for 3D structure formation, we can only speculate on their possible physiological role in the context of ovarian cancer cell spheroids based on the necessity of 3D organization for survival, proliferation and metastasis. The experimental model, i.e., aggregation of diverse EOC cell line in 3D structure, confirmed that αSMA is neo-expressed and thus is likely crucial for the compaction and survival of the ovarian cancer cells as 3D structures.

On the other hand, FN1 might also constitute an extracellular substrate for ovarian cancer cell aggregation in spheroids in the absence of stromal fibroblasts. It had been previously demonstrated that soluble ECM proteins, such as FN1 and vitronectin are detectable in ascitic fluid [28] and are organized by integrins for survival and proliferation (for a review see [44]). The role of endogenous FN1 in metastasis has been previously demonstrated in experimental models of ovarian cancers. Using in vitro reconstituted models, Kenny et al. [19] and Iwanicki et al. [11] showed that FN1, either secreted by mesothelial cells or by EOC cells themselves, is necessary to allow 3D structures formed by ovarian cancer cells to survive in the absence of anchorage and in an unfitting metabolic environment. Moreover, silencing of its receptor, integrin α_5_ [13], and antibodies blocking FN1 and α_5_ or β_1_ integrin function prevented the formation of experimental metastases [19].

## 5. Conclusions

Data demonstrate that multicellular aggregates found in ovarian cancer ascites are made almost exclusively by cancer cells, thus reinforcing the assumption that they are responsible for peritoneal metastasis of ovarian cancer. Data also show that aggregates display a hybrid phenotype that allows maintenance of the polarized 3D structures, necessary for survival and proliferation of cancer cells, but also the plasticity necessary for seeding into peritoneal lining.

## Supplementary Materials

The following are available online at https://www.mdpi.com/article/10.3390/ijms23020833/s1.
