# Supplementary material for: Ovarian Cancer Cells in Ascites Form Aggregates That Display a Hybrid Epithelial-Mesenchymal Phenotype and Allows Survival and Proliferation of Metastasizing Cells
Source: Int J Mol Sci. 2022 Jan 13;23(2):833. doi: 10.3390/ijms23020833 (PMC8775835; doi:10.3390/ijms23020833)
Supplement: Supplementary file 1 [file ijms-23-00833-s001.zip › Figure S1.pdf]

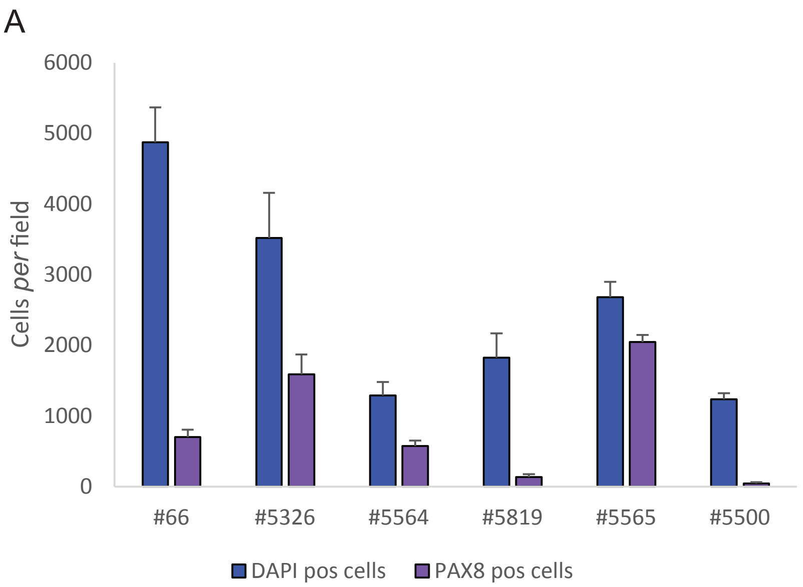

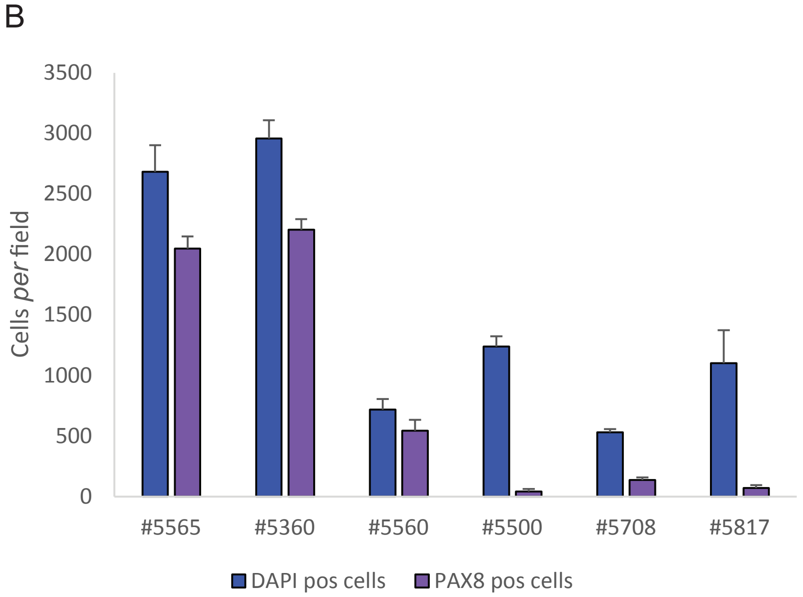

Supplementary Figure S1: Quantification (see Methods section) of HGS-EOC cells in the bulk ascites samples, based on PAX8+ staining, compared to the total number of cells/field based on DAPI staining. Numbers on correspond to the Profiling protocol numbers of the collected ascites. Details of each #case are in Supplementary Table 1. (A): six independent cases; (B) comparison of three successive withdrawals of ascites of the same two patients (details in Supplementary Table 1).
