# Supplementary material for: Ovarian Cancer Cells in Ascites Form Aggregates That Display a Hybrid Epithelial-Mesenchymal Phenotype and Allows Survival and Proliferation of Metastasizing Cells
Source: Int J Mol Sci. 2022 Jan 13;23(2):833. doi: 10.3390/ijms23020833 (PMC8775835; doi:10.3390/ijms23020833)
Supplement: Supplementary file 1 [file ijms-23-00833-s001.zip › Figure S2.pdf]

A B

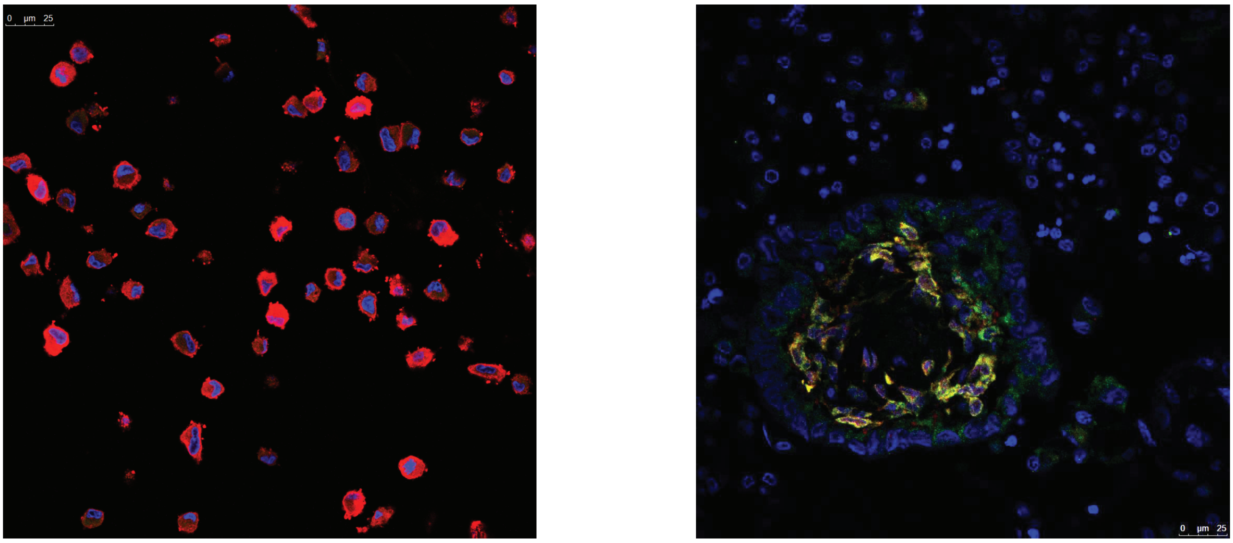

Supplementary Figure S2: Control staining to assess the specificity of αSMA MAbs. (A) IF staining of commercially available hu-man fibroblasts (BJ-5ta CRL-4001™) with αSMA MAbs conjugated with AlexaFluor ®555. (B) Staining of ascites spheroids with the same αSMA and with a different primary MAbs followed by secondary an-ti-mouse MAb conjugated with Alexa Fluor®488. Nuclei were stained with DAPI.
