# Supplementary material for: Ovarian Cancer Cells in Ascites Form Aggregates That Display a Hybrid Epithelial-Mesenchymal Phenotype and Allows Survival and Proliferation of Metastasizing Cells
Source: Int J Mol Sci. 2022 Jan 13;23(2):833. doi: 10.3390/ijms23020833 (PMC8775835; doi:10.3390/ijms23020833)
Supplement: Supplementary file 1 [file ijms-23-00833-s001.zip › Figure S3.pdf]

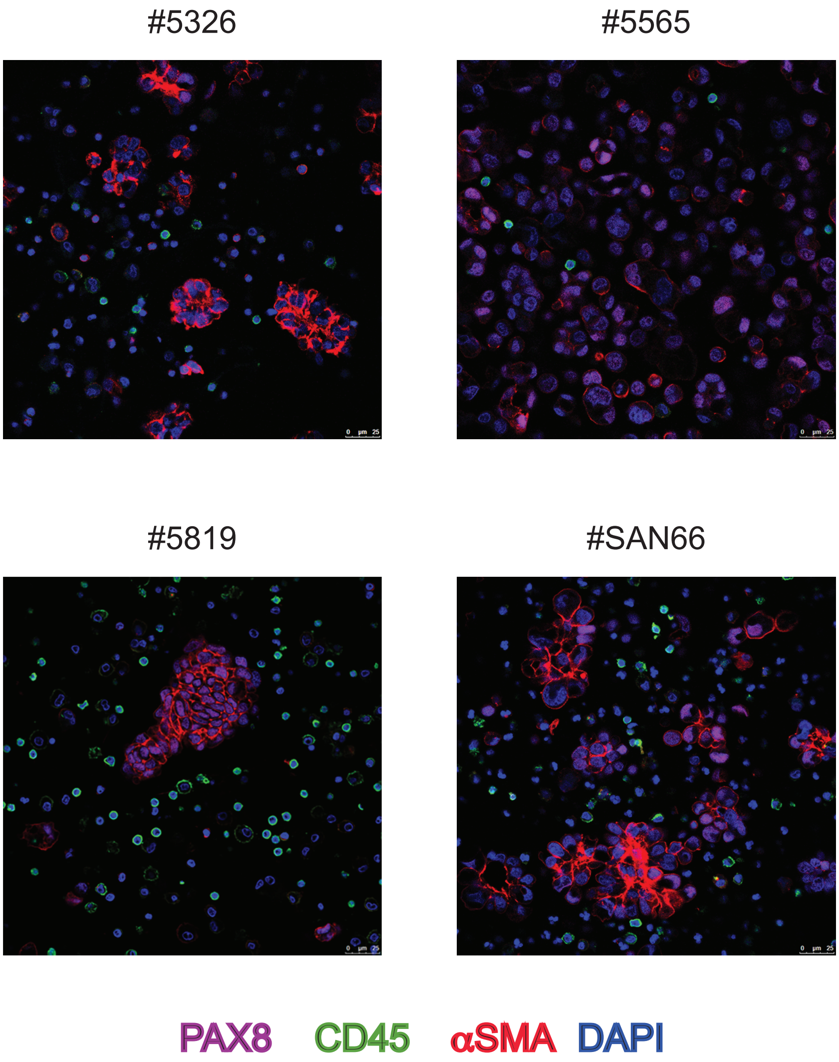

Supplementary Figure S3: IF staining of bulk ascites sections to show CD45 positive cells.
