# Supplementary material for: Ovarian Cancer Cells in Ascites Form Aggregates That Display a Hybrid Epithelial-Mesenchymal Phenotype and Allows Survival and Proliferation of Metastasizing Cells
Source: Int J Mol Sci. 2022 Jan 13;23(2):833. doi: 10.3390/ijms23020833 (PMC8775835; doi:10.3390/ijms23020833)
Supplement: Supplementary file 1 [file ijms-23-00833-s001.zip › Figure S4.pdf]

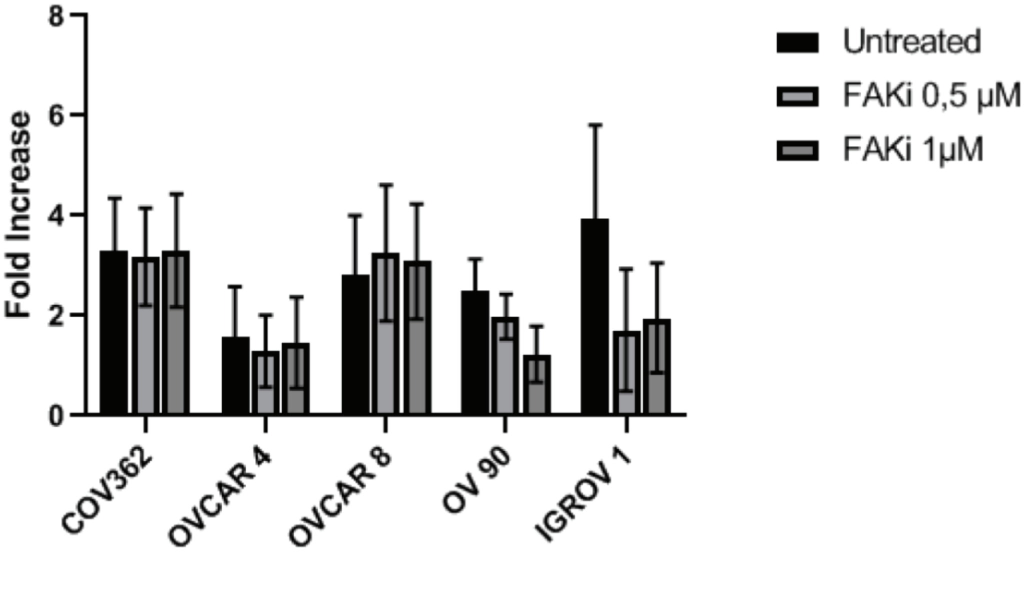

Supplementary Figure S4: CellTiterGlo assay to measure the proliferation of the listed ovarian cancer cell lines in the presence of the PDN-1186 FAK inhibitor (FAKi) at the indicated concentrations.
