# Supplementary material for: Ovarian Cancer Cells in Ascites Form Aggregates That Display a Hybrid Epithelial-Mesenchymal Phenotype and Allows Survival and Proliferation of Metastasizing Cells
Source: Int J Mol Sci. 2022 Jan 13;23(2):833. doi: 10.3390/ijms23020833 (PMC8775835; doi:10.3390/ijms23020833)
Supplement: Supplementary file 1 [file ijms-23-00833-s001.zip › Table S1.pdf]

Table S1: clinical and pathological information of six patients analyzed

	A	B	C	D	E	F	G	H	I
1	**Clinical/patient information**				**Clinical/tumor information**				
2	**patient ID**	**ascites ID/profiling no.**	**age**	**Alive/died of disease (DOD)/ lost**	**histology**	**Tumor grade classification**	**FIGO stage**	**Is ascites from untreated patient?**	**chemotherapy: lines of treament**
3									
4	1	#5326/#5421	80	DOD	high-grade serous	Grade 3	IIB	YES/NO	Carboplatin/paclitaxel
5	2	5564	69	Alive	high-grade serous	Grade 3	IIIB	NO	Carboplatin/paclitaxel
6	3	#5565/#5360/#5560	73	DOD	high-grade serous	Grade 3	IV	NO	Carboplatin/paclitaxel
7	4	#5500/#5708/#5817	58	DOD	high-grade serous	NA	IV (clinical)	NO	Carboplatin-Paclitaxel (first line); Caelyx (second line)
8	5	#5819	64	Alive	high-grade serous	Grade 3	IV	YES	Carboplatin/paclitaxel
9	6	SAN66	68	DOD	clear cell	Grade 3	IV (clinical)	NO	Carboplatin/paclitaxel
